# Collaborative partnership and the social value of clinical research: a qualitative secondary analysis

**DOI:** 10.1186/s12910-017-0217-6

**Published:** 2017-10-25

**Authors:** Sanna-Maria Nurmi, Arja Halkoaho, Mari Kangasniemi, Anna-Maija Pietilä

**Affiliations:** 1 Department of Nursing Science, University of Eastern Finland, Kuopio Campus, P.O. Box 1627, 70211 Kuopio, Finland; 2 Kuopio University Hospital (Science Service Centre), P.O. Box 100, FI 70029 KYS Kuopio, Finland; 3 Social and Health Care Services, Kuopio, Finland

**Keywords:** Clinical research, Collaborative partnership., Deductive-inductive content analysis., An Ethical Framework for Biomedical Research., Hospital., Management., Secondary analysis., Social value.

## Abstract

**Background:**

Protecting human subjects from being exploited is one of the main ethical challenges for clinical research. However, there is also a responsibility to protect and respect the communities who are hosting the research. Recently, attention has focused on the most efficient way of carrying out clinical research, so that it benefits society by providing valuable research while simultaneously protecting and respecting the human subjects and the communities where the research is conducted. Collaboration between partners plays an important role and that is why we carried out a study to describe how collaborative partnership and social value are emerging in clinical research.

**Methods:**

A supra-analysis design for qualitative descriptive secondary analysis was employed to consider a novel research question that pertained to nurse leaders’ perceptions of ethical recruitment in clinical research and the ethics-related aspects of clinical research from the perspective of administrative staff. The data consisted of two separate pre-existing datasets, comprising 451 pages from 41 interviews, and we considered the research question by using deductive-inductive content analysis with NVivo software. A deductive analysis matrix was generated on the basis of two requirements, namely collaborative partnership and social value, as presented in An Ethical Framework for Biomedical Research by Emanuel et al.

**Results:**

The findings showed that collaborative partnership was a cornerstone for ethical clinical research and ways to foster inter-partner collaboration were indicated, such as supporting mutual respect and equality, shared goals and clearly defined roles and responsibilities. In addition, the social value of clinical research was an important precondition for ethical clinical research and its realisation required the research partners to demonstrate collaboration and shared responsibility during the research process. However, concerns emerged that the multidimensional meaning of clinical research for society was not fully recognised. Achieving greater social value for clinical research required greater transparency, setting research priorities, shared responsibility for the dissemination and use of the findings and stronger community awareness of the ethics-related aspects of clinical research.

**Conclusions:**

Collaborative partnership and social values are essential for protecting the human subjects and communities involved in clinical research.

## Background

Protecting human subjects from being exploited is one of the main ethical challenges for clinical research [1]. However, there is also a responsibility to protect and respect the communities who are hosting the research [2–5]. Recently, attention has focused on the most efficient way of carrying out clinical research so that it benefits society by providing valuable research, while simultaneously protecting and respecting the human subjects and the communities where the research is conducted [5–7]. Collaboration between partners plays an important role here [2].

Emanuel et al. [2, 3] have presented requirements for collaborative partnership and social value, which focus more on the public and social benefits. These authors have shown that a collaborative partnership needs to be established between the researchers and the community in which the clinical research is being conducted [2]. Earlier literature also recognised that collaboration between research partners was an important element of successful and ethical clinical research [6, 8–12]. Collaborative partnership between researchers, communities, health policy makers, and research sponsors help to ensure that the clinical research that is carried out is acceptable, is responsive to the community’s actual health problems and provides worthwhile benefits to the community. If the community agrees to take part, this can help to avoid exploitation and makes it possible to respect the community’s values, circumstances, culture and social practices [2]. In collaborative partnership, information on the responsibilities and privileges related to the research is distributed more widely in the community [2, 6, 8, 9]. Each partner plays its own separate role in ensuring and maintaining the ethical conduct of the clinical research [13] and each offers distinct instruments and support that contribute to the foundation for ethical clinical research [13–16]. For example, senior hospital managers are responsible for providing a research environment and organisational culture that enables high-quality research within their respective organisations [11, 16]. Meanwhile, researchers bear primary responsibility for developing a scientifically valid research protocol and for safeguarding and overseeing the health, wellbeing and rights of the subjects during research projects [14]. Finally, clinical results are unlikely to have a lasting impact on policy making or the allocation of scarce resources without the commitment and investment of those responsible for making health policies [2].

To be ethical, clinical research must have social value [2, 3], which means that it must be beneficial to its human subjects, the community and society at large [2–4]. A lot of the international and national regulations and research ethics literature state that clinical research is ethically acceptable when it has social value [17, 18]. Despite the widely recognised fundamental importance of social value, this ethical requirement remains a seldom-studied phenomenon [4, 17–20] and no systematic analyses exist on why social value is an ethical requirement [20]. The justification for clinical research involving human subjects is related to its ability to produce scientific knowledge that can be used to improve the public’s health and wellbeing without wasting resources [2, 3, 17, 18]. The social value of a clinical research study must be sufficient to justify the risks and burdens of the research for research participants and others who may be affected [4, 19]. If clinical research fails to generate social value, it exposes human subjects to risks and burdens without achieving any valid research findings [2, 3] and wastes limited resources. Because most clinical research is carried out with the aid of public funding, how these scarce resources are used is of significance [2, 21, 22].

The social value of clinical research is an important requirement for decisions makers [19]. It is also important for those who evaluate the ethical aspects and acceptability of the proposed clinical research, such as research site managers and ethics committees who are charged with protecting the rights and wellbeing of research participants and their organisations [4, 19]. It is also important for health research policy makers and researchers who make decisions about what scientific topics to pursue. [4, 19]. Public officials, such as administrative managers, also have an obligation to promote the use of public resources in a way that promotes socially valuable goals [20].

In summary, collaborative partnership are an important part of successful and ethical clinical research [2, 11, 12]. Despite this, little attention has been paid to identifying how collaborative partnership occur and what are required for them to be effective in clinical research practice In addition, we need more empirical literature on how ethical research values are understood and applied by key stakeholders in research [23]. For those reasons, the aim of this research was to find out how collaborative partnerships and social value are presented in clinical research, from the point of view of nurse leaders and administrative staff, such as principal investigators, administrative managers and elected officials**.**

## Methods

### Study design

**Fig. 1:**
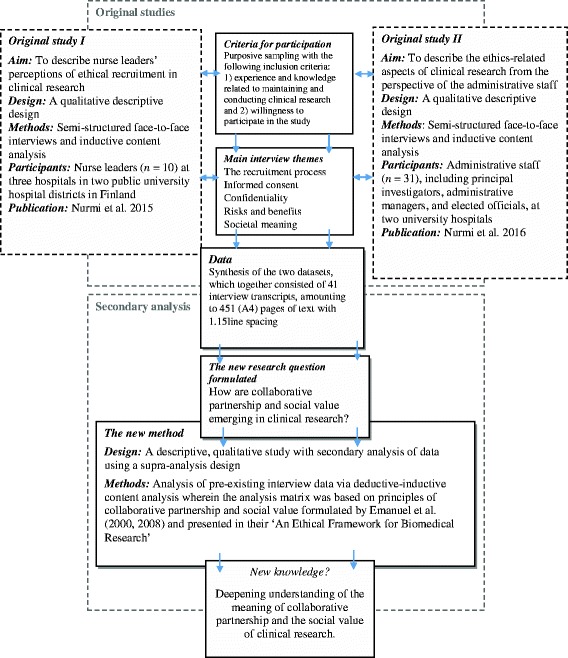
Study design

A qualitative secondary supra-analysis was conducted that employed two pre-existing sets of interview data [24, 25] that had formed the basis of previously published study reports by the lead author of this paper [11, 12]. Secondary analysis was chosen for this descriptive work, because it enabled us to focus on what had jointly emerged from the two datasets: the combination of datasets provided an opportunity to gain a broader and deeper conceptual understanding of the research phenomenon [24, 25] and highlighted previously unreported results [25, 26]. A new empirical research question was formulated on the basis of the data from the two primary studies [24, 25]. This pertained to the perceptions of ethical recruitment in clinical research by nurse leaders [12] and to the perceptions of the ethics-related aspects of clinical research by administrative staff [11] (Fig. 1). These stakeholders have major professional responsibilities for enabling and managing the conduct of clinical research in their hospitals, as described in original studies [11, 12]. Despite this, their common perspectives in relation to the ethical aspects of clinical research have seldom been studied.

### Data

The combined body of data for the secondary analysis came from 41 transcripts of face-to-face personal interviews conducted in 2012–2014 (Fig. 1) [11, 12]. The themes of these semi-structured interviews were based on previous literature and the content was related to the recruitment process, informed consent, confidentiality, risk and benefits and societal meaning [27–29]. The same themes, with some minor changes in context, were used for all participants [30]. The interviews, which lasted 42 to 78 min, were audio-recorded, then transcribed verbatim. The material amounted to 451 A4 pages of text in Times New Roman size 12 with 1.15 line spacing.

### Data analysis

**Fig. 2:**
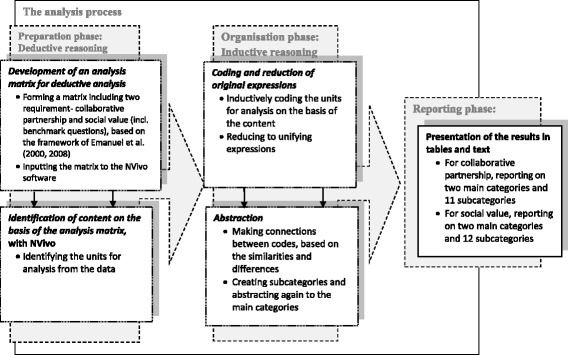
The analysis process

The deductive-inductive content analysis took place in three phases [31] and these are depicted in Fig. 2. The first phase was to create the deductive analysis matrix on the basis of two requirements, collaborative partnership and social value, and the accompanying benchmarks, as laid down in An Ethical Framework for Biomedical Research by Emanuel et al. [2, 3] This framework was chosen because it highlighted the important meaning of collaborative partnership [2], which was also found to be a prerequisite for ethical conduct of clinical research in our pre-existing interview data [11, 12]. In addition, this framework contained requirement of social value [2, 3], whose fundamental importance has been well recognised, but seldom studied [4, 17–20]. In this study, the framework provided a systematic and clear structure that was suitable for addressing our research question and identifying central ethical aspects, questions and concerns related to clinical research from the perspective of our participants.

The matrix was then created and inputted into NVivo analysis software, version 10 [32]. This consisted of requirements for collaborative partnership and social value, which were both specified in relation to benchmarks that highlighted practical considerations related to implementation. Then the transcripts were read through several times to generate an understanding of the dataset as a unified entity. After that, the NVivo matrix was used to identify units for analysis, consisting of phrases, sentences or thoughts. The second phase, which was an inductive one, entailed organising the data. The units were coded separately, but inductively, in line with the similarities and differences in content and again connected to subcategories and then abstracted to main categories. During the third phase, which was reporting, the results were presented in tabular and text form. [31] Our analysis identified two main categories and 11 subcategories that referred to recruiting the collaborative partnership and two main categories and 12 subcategories were identified that referred to the requirement for social value, (Table 1).

**Table 1:** The categories identified in the analysis

Subcategory	Main category	Requirement
Value-based collaboration with human subjects	Collaboration by partners as a cornerstone of ethical clinical research	Collaborative partnership
Multi-profession collaboration with the research site/organisation		
Collaboration and commitment to common goals in multi-centre research		
Collaboration that involves dialogue with an ethics committee		
Patient organisations as an important element linking patients, health-care professionals, and researchers		
Mutual collaboration between researchers and makers of health policy		
Mutual respect and equality between partners	Ways of fostering collaboration between partners	
Fair benefits to the research site for participation		
Active involvement of researchers, along with regular contact and joint meetings		
Shared goals and clearly defined and recognised roles and responsibilities for the partners		
A collaborative organisational culture		
Generation of scientific knowledge	Clinical research’s multidimensional but not fully recognised meaning	Social value
Improvements in infrastructure		
Well-trained and competent clinical staff and researchers		
Value for the local economy		
High-quality medical education and advancement of the scientific process		
Methods for evaluating the impact and quality of medical treatment		
The partly unrecognised meaning of clinical research		
Shared responsibility to plan and execute socially valuable clinical research	Ways to increase social value: shared responsibility	
Greater transparency of clinical research		
Responsible and transparent dissemination of research findings and implementation in clinical practice and health-related policymaking		
Fairness in setting of future research priorities		
Increased community awareness surrounding the ethics-linked aspects of clinical research		

## Results

On the basis of our secondary supra-analysis, the participants’ views about the collaborative partnership were divided into two main categories (Table 1): collaboration by partners as a cornerstone of ethical clinical research and ways of fostering collaboration between partners. In the second category, the section covering social value was grouped into two main categories: i) the multidimensional but not fully recognised meaning of clinical research and ii) ways to increase the shared responsibility for social value:

### Collaborative partnership

#### Collaboration by partners as a cornerstone of ethical clinical research

The participants felt that the collaboration of the partners was a cornerstone for respecting human subjects and communities in clinical research. They stated that the role of collaboration in bringing about ethical clinical research had increased in response to the evolving research environment. In addition, they emphasised that collaboration between various partners, such as researchers, senior hospital managers, clinicians, collaborators from various organisations and countries and those who drive health policy, was needed at all stages of clinical research. These ranged from selecting an important research topic to disseminating and using the research results in healthcare practice and health-related policy making.

The participants described value-based collaboration between researchers/clinicians and human subjects*,* which were built on the values of trust, individuality, respect, honesty, equality and cultural angles. For example, professional consideration of a subject’s individuality and cultural factors during the disclosure of information was deemed a prerequisite for autonomous informed content. In addition, participants cited multi-profession collaboration between researchers and hospital staff, such as site managers, clinicians, and research nurses, as a factor that led to better ethics in clinical research. For instance, they concluded that a planning process that involved professionals with diverse backgrounds, values and expertise made it possible to gain a more comprehensive and holistic view of the ethical aspects of the research process, such as the recruitment of human subjects.

*“In the planning phase, we invite all important partners, such as the research group, nurse leaders and partners from other research units, to a meeting, where we plan, for example, how to implement research in practice. It is important to consider all diverse perspectives, because if this is neglected, we will fail in our goal. The most challenging research projects are those involving many different wards and/or research organisations” (interview 30).*

Moreover, the participants stated that collaboration in multi-centre research and research involving many research sites should be based on a common understanding and commitment to shared goals and values. For example, they considered it important that partners in multi-centre research openly discussed the ethics-related aspects of the research project, agreed on common practices in research implementation and committed to the associated compliance. However, dialogue-based collaboration between ethics committees and researchers was also seen as a key prerequisite for ethically implemented clinical research, because it led to more in-depth consideration of the ethical aspects of research and deeper ethical reflection. Furthermore, participants stated that patient organisations were important partners, because they provided a valuable link between patients, healthcare professionals and researchers. For example, patient organisations had provided valuable information about a patient’s health needs, improved the recruitment of human subjects by informing target groups about forthcoming research and improved the dissemination and use of research findings in the area of public health. In addition, participants described the importance of mutual collaboration between researchers and makers of health policy as one of the prerequisites for, among other things, devising health-related policies that were informed by research findings. However, their views were that this collaboration was insufficient and that the barriers to this collaboration included a lack of joint meetings, lack of commitment to promoting collaboration and differences in culture and the basis/objectives for scientific research and developing health policies.

*“How could we get connections to health policy makers, so that we could tell them our message? Dialogues between the practical level and health policy makers is limited. Thus we should take a more active role in this, because there are so many things that we could influence through clinical research, but at the moment we don’t have that kind of collaboration or encounters” (interview 33).*

#### Ways of fostering collaboration between partners

The participants described five ways to foster collaboration among partners. The first was mutual respect and the equality of partners. One key issue cited in connection with this was that the prevailing circumstances, practices and values of the research sites should be respected during the research activities, for example planning clinical research as an integral part of the sites’ daily routines in collaboration with site staff. The second issue was that the implementation of any research findings need to provide worthwhile benefits for the research site in return for their participation in the research. For instance, research findings that could be used to improve clinical practice and staff competence were seen as worthwhile benefits of participation. Thirdly, participants stated that workable collaboration between partners required researchers to get actively involved and this included the necessity for regular contact and joint meetings. For example, it was important that meetings and other contact was planned, scheduled in a timely manner in the critical phase of the research process and provided an atmosphere that enabled dialogue and a two-way flow of honest feedback. A fourth key issue was shared goals and clearly defined and recognised roles and responsibilities for each partner during the research process. It was important for the partners to recognise their roles and responsibilities during that process and to be well educated with regard to the research project and committed to carrying out their research-related duties.

*“Well, it is important that we have mutual understanding about our roles and responsibilities during the research, we have shared goals and that we are all committed to implementing research right from the beginning of the process” (interview 8).*

The fifth factor for enabling collaboration between partners was an organisational culture that encouraged collaboration and created opportunities for networking. For example, the management’s role in creating such a culture was seen as indisputable.

### The social value of clinical research

#### Clinical research’s multidimensional but not fully recognised meaning

The clinical research was seen as highly valuable, producing benefits for human subjects, patient groups, the public, clinicians, the hospitals hosting the research, healthcare organisations and society in general. The meaning and outcomes of the clinical research were described in terms of six dimensions. The first dimension was the scientific knowledge generated, which led to, for example, improvements in public health and medical care, while also promoting health equality and providing a basis for coherent and evidence-based healthcare practice. The second dimension was improvements in the research and care infrastructure in hospitals. For example, clinical research generated research resources, such as money and equipment and provided early access to information, such as new diagnostic methods and treatments and access to novel health technology. The third dimension involved well-trained and competent clinical staff and researchers. For example, our participants said that the clinical staff involved in clinical research were trained to provide the best possible care to patients and that they were also more competent to carry out research-related tasks in an ethical way. The fourth dimension was the value that clinical research provided for the local economy, such as creating new jobs and innovation, bringing corporate tax revenues to the region and reducing healthcare costs by identifying more effective treatments or enhancing the population’s health. The fifth dimension was high-quality medical education and advancing the scientific process. For example, clinical research has produced scientific knowledge that has provided a foundation for high-quality medical education and innovations and better outcomes have followed due to the international and multidisciplinary nature of clinical research,. The final dimension was methods that evaluated the impact and quality of medical treatment, allowing, for example, a fairer allocation of scarce healthcare resources.

*“The societal value of clinical research is very significant. Clinical research improves medical care, promotes populations’ health, creates new, better and more effective treatments and, at the same time, it maintains researchers and clinicians know-how. In addition, there is this educational aspect, as clinical research provides the basis and conditions for high-quality medical education. Yes, and, of course, clinical research has this commercial-industrial significance” (interview 29).*

However, participants expressed a concern that the multidimensional meaning and value of clinical research was sometimes unrecognised by society, for example among those responsible for health policies. Furthermore, they stated that demonstrating and measuring the benefits and value of clinical research was insufficient and that the nature of research activities at university hospitals was somewhat unclear from an external perspective, as the benefits and value of clinical research had not been explained sufficiently to some stakeholders, such as policymakers.

*“The important value of clinical research is not fully recognised. The clinical research is subjected to enormous saving pressures, and, for example, all decision-makers don’t understand how much investing in clinical research would benefit our society” (interview 16).*

#### Ways to increase social value: Shared responsibility

The participants considered the social value of clinical research as an ethical requirement for clinical research. They described the shared responsibilities of researchers, research site managers, clinicians and patient organisations, to plan and recognise socially valuable research topics. In addition, researchers and managers in research organisation shared the responsibility for assessing the importance and value of the study in collaboration with their ethics committees. For example, it was seen as important that when site managers gave their approval for research they assessed whether the researchers were responsible for study sites and patients’ health problems and whether the research would produce benefits for the research site, patient group and society.

The participants demanded more transparent and detailed presentation of clinical research activity, its impact and the benefit that clinical research would yield for healthcare, the economy and society in general. In their opinion, the researchers and senior hospital managers had shared responsibility for promoting the transparency of clinical research. For example, the participants called for clarification of what kind of research was conducted, who had funded it, the kind of research findings obtained, the impact of the research, how the findings had been used in clinical practice and health-related policy making and the benefits, including financial ones, relative to the costs. However, demonstrating and measuring the impact and benefits of clinical research was acknowledged to be extremely difficult. This led to demands for new forms of collaboration and for procedures for evaluation and reporting that were systematic and standardised.

*“The significance and effectiveness of clinical research should be presented more clearly. Research groups should demonstrate the results and effectiveness of the study in practice But the effectiveness and significance of the overall clinical research activity is attributed to leading hospital managers and officials” (interview 40).*

The participants’ experience was that the dissemination of clinical research findings to beneficiaries, such as patient groups, the general public and policy makers, had often been insufficient, non-systematic and uncontrolled. For example, plans about how to disseminate the findings were often lacking, with researchers bearing sole responsibility for this. In addition, organisations lacked clear guidance and information was not reported using multiple channels. Thus, our participants said that, social value would only be achieved if there was responsible and transparent dissemination of the research findings and that they were translated into clinical practice and health policies. The key issue cited was that the researchers and the collaborating hospital hosting the research and its managers shared responsibility for disseminating the findings and using them in clinical practice and policy making. For example, more effective dissemination of the results required detailed planning by the partners, the hospitals needed to support the researchers to disseminate the findings, through education and access to appropriate dissemination channels and contacts, and dissemination activities needed to be monitored.

*“It would be important that hospitals hosting clinical research would guide researchers to wider dialogue and communication of research results outside the hospitals, such as for health policy makers. Hospitals should provide support for researchers related to research dissemination and leading hospital managers should try to help researchers to collaborate with health policy makers” (interview 11).*

According to the participants, one key way to increase the social value of clinical research was by setting future research priorities in such a way that limited research and healthcare resources were allocated. This would produce the maximum value to society and use healthcare resources fairly. They stated that clearly defined, transparent priorities helped researchers, funders, hospitals hosting research, ethics committees and others to direct resources fairly to the research that offered the greatest potential benefits to the public and to health. The participants said that future research priorities should be based on discussions about both values and scientific knowledge.

*“Setting research priorities should receive more attention. How do we allocate scarce resources fairly and in an equal manner? Treatments and medication become more and more expensive and we can’t afford everything. It is important that clinical research producing social value is executed in the most effective manner and we should allocate resources to research that produce more social value and increases populations’ health. At the moment, this is not so in all cases” (interview 15).*

Greater community awareness surrounding the ethics-related aspects of clinical research was seen as another element that increased public trust and, through that, the social value of clinical research. For example, this could increase the public’s trust in clinical research, have positive effects on the recruitment of human subjects and their commitment to research processes and enable better use of the research findings in relation to the public’s health-related behaviour. The responsibility to increase discussions about the ethics of clinical research at a societal level was seen as resting with researchers, senior hospital managers and research ethics committees.

## Discussion

The findings from our study emphasise that collaborative partnership and social value are important requirements when it comes to protecting and respecting human subjects and communities in clinical research. Our findings show that collaborative partnership can be seen as one central cornerstone of ethical clinical research. The responsibilities for ethical clinical research were found to be shared among the partners, such as researchers, human subjects, clinicians, senior hospital managers, ethics committees, patient organisations and policymakers. Emanuel et al. [2] also recognised that these extended throughout the wider social context. These partners play various distinct roles in the clinical research process, with regard to ensuring and maintaining ethical conduct during clinical research [13] and they differ in terms of the instruments and support they offer for the foundations of ethical clinical research [13–16].

The participants in this study described collaboration and how it varied between different partners, such as value-based collaboration between researchers and clinicians and human subjects or mutual collaboration between researchers and those who devise health policies. However, there remains a widely known gap in the collaboration between policymakers and researchers in the health field [21, 22, 33], which was also identified by our participants. This gap may have a significant influence on, for example, how research findings inform health-related policy making, the dissemination of research findings, unmet health needs and the allocation of resources [33, 34]. The gap may also lead to wasteful or even unnecessary medical research [21, 35, 36]. The results of our study point to a need to strengthen the collaboration between partners, especially between health sector policy makers, researchers and the senior managers at hospitals, in order to guarantee ethical clinical research.

One crucial question that needs to be addressed is how we can foster collaboration between partners. Our participants emphasised that key factors that may foster collaboration between partners included mutual respect and equality among the partners, worthwhile benefits for the research site in exchange for participating in the clinical research, the active involvement of researchers - including regular contact and joint meetings - ensuring shared goals and clearly defined roles and responsibilities for the partners and an organisational culture that encourages collaboration and creates opportunities for networking. These factors, alongside strong professional leadership, could result in better outcomes for clinical research and they should be considered more fully in collaborative research activity [37].

The results of our study also indicate that the participants considered that the social value of clinical research was an important precondition for ethical clinical research and that it was a prerequisite that needed to be realised through the research process. The realisation of this objective required collaboration and shared responsibility by the research partners. Many ethical guidelines and previous literature [4, 17, 18, 20, 38], in line with Emmanuel et al. [2, 3], has stated that social value is an ethical requirement of clinical research. Despite this, social value is rarely studied in this context [4, 17, 18, 20] and no systematic analyses exist to explain why social value is an ethical requirement [20]. Habets et al. [17] recognised that the concept of social value was ambiguous by nature and uncertainty exists as to how social value can be defined, actualised and evaluated during the research process. Some authors have argued that the requirement for social value is not persuasive and that, in some cases, it was acceptable to conduct clinical research that was known to have no social value [39, 40]. It is evident that there is a need to examine social value as a concept and ethical requirement in greater depth [23], from the perspectives of different research partner.

The participants in this study described the multidimensional meaning and value of clinical research for society. In agreement with Emanuel et al. [2, 3], they described the instrumental value of knowledge gained from clinical research, leading to improvements in public health, wellbeing and healthcare. However, alongside the instrumental value, they pointed out that clinical research has been shown to have intrinsic value in its own right [41]. This encompassed elements such as improvements in the research and care infrastructure and in clinicians’ competence with respect to research-related issues. According to Wendler and Rid [20] the standard perception of social value does not exclude the possibility that clinical research may be socially valuable in ways that do not directly lead to improvements in health, such as creating employment, but these should not be the priority. Furthermore, our study found that the multidimensional meaning of clinical research was not fully recognised in our society: the actions involved were partially unclear and the benefits or value of research had not been introduced to key partners such as health sector policy makers. Because a large proportion of clinical research was implemented with public funding, it was significant how these thinly spread resources are used [2, 3, 21]. Also, the benefits and value of clinical research needed to be demonstrated in detail in order to engage and maintain the necessary support for clinical research at all levels, including the ethical and financial elements [42]. The requirement for clinical research to offer social value helped to ensure the proper stewardship of public resources [20].

Our participants highlighted that the researchers and the senior hospital managers of had a responsibility to demonstrate the benefits and value gained from clinical research in their hospitals, in particular to policy makers and the public. Continuing concerns related to poor-quality and wasteful medical research have drawn attention to the need for increased transparency and quality improvements in clinical research, a point that was also identified by our participants. As noted in previous studies, if clinical research is to be ethical, its impact and the value it adds to both the healthcare system and society must be efficiently determined [2, 5]. However, it is known that such action is extremely challenging and there is little clear empirical evidence of how the impact of clinical research can be operationalised and measured in a standardised manner [42–44]. Therefore, the medical research community, public funding agencies, healthcare professionals and senior hospital managers should work together to develop more robust methods for identifying and describing the impact of clinical research [21, 35, 36, 43].

Our findings point strongly to the need for researchers and the senior hospital managers to share the responsibility for disseminating research findings and using these to inform clinical practice, health-related policy making, and public health actions. This was considered important, because our study found that researchers had to shoulder the responsibility for disseminating the findings on their own. The social value of clinical research can only be realised if the findings are translated into health improvements and advances in healthcare and clinical practice and used to create health policies [2, 3]. However, according to the participants in our study, as well as earlier studies, using and disseminating clinical research findings in healthcare practice and policymaking was a highly complex process [2, 45, 46], and translating research findings into health benefits could be very challenging [2, 47]. In common with the result of earlier studies, our participants stated that current efforts to disseminate the findings of clinical research and put them into use were inadequate [45–48]. More support from organisations that hosted clinical research was also required and this included assistance with researchers’ education, access to suitable dissemination channels, help in establishing new contacts and networks, clear guidance and monitoring of dissemination techniques. The results of our study indicated that senior hospital managers had an important responsibility to create opportunities for more fruitful co-operation and dialogue between researchers and health sector policy makers, along with actively sharing research findings with those policy makers.

Recent literature has provided a link between social value requirements and debates about setting research priorities [19, 23]. For example, Barsdrof and Millum [19] argued that the social value of health research should be conceptualised as a function of the expected benefits of the research and the priority that the beneficiaries deserve. In their view, this conception of social value requires that certain types of research should be prioritised in order to benefit the world’s poorest citizens [23]. Our participants also described this link between the social value requirement and setting research priorities and also mentioned the challenges related to setting future research priorities, with regard to ensuring that scarce research and healthcare resources were allocated fairly and that the maximum value was generated for society. Our participants felt that clearly defined, transparent priorities would help researchers, funders, the hospitals hosting research, ethics committees and others to direct resources fairly to the research that offered the greatest potential public health benefits. They also stated that diverse views should be heard when value choices of this nature were being made.

Clinical research provides significant benefits to society and generating and preserving these benefits depends on the public’s trust in clinical research [23] and the support they give it [1, 23]. When the social value of clinical research is realised, potential research participants can be sure that they will only be invited to face the risk and burdens of clinical research when the study has the potential to improve health [23]. Allowing clinical research to be carried out that has no social value may undermine the public’s trust in, and support for, clinical research [20]. Our participants said that one way of increasing social value and public trust in clinical research was through increased community awareness about clinical research and the ethics-related aspects of that research. In agreement with earlier literature, our participants recognised that the public had limited knowledge about clinical research [49–52], and that they needed a better understanding of, and greater education about, clinical research practices, the importance, meaning and benefits for society and the ethical angles [1, 7, 49, 53]. Making clinical research a part of the everyday landscape of our society has been identified as an important goal [50] and our participants also highlighted this. So is increasing both the transparency of the research process [49] and the knowledge that the public and policy makers have of research activity, along with the value and benefits of clinical research.. Institutions that conduct clinical research must cultivate and maintain open interaction with the local community with regard to research activities and the protection of human subjects [13, 53]. We argue that making the public and the health policy sector more aware of the ethical issues related to clinical research should be given a high priority.

### Methodological considerations and limitations

Our secondary supra-analysis contributes to a previously limited body of knowledge and provides a broader and deeper conceptual understanding [25] from the perspective of nurse leaders and administrative staff, which is an issue that has seldom been studied [11, 12]. One limitation of this study was that the data that were used were not collected specifically for our study, so it is possible that the data were distorted during the secondary analysis [33]. However, the trustworthiness of this study was supported by the fact that both of the original studies were underpinned by the same research tradition [30, 31] and that they used the same data collection and analysis methods [30, 54]. In addition, they were both performed by the first author of this paper. The author’s expertise and knowledge of the primary data ensured that the results of our secondary supra-analysis were true to the data [30]. The pre-existing, interview-based dataset proved to be rich and detailed. Also, saturation was achieved and the data were well suited to answering the new research question that emerged from the two previous studies [11, 12]. Another limitation of this study is that the results do not present similarities and differences between the different participant groups, namely nurse leaders and administrative staff. We chose to analyse the data as whole in order to protect the privacy and confidentiality of our participants, because the number of participants in the specific participant groups was relatively low. Furthermore, our aim was to explore the phenomenon at the conceptual level and as a whole from the perspective of those who had responsibilities for conducting and maintaining clinical research in their hospitals. In the future it is important to examine research partners perspectives separately, because the social value is likely to be conceptualised and measured differently by different beneficiaries of clinical research [22].

## Conclusions

Collaborative partnership is one of the cornerstones of ethical clinical research. Therefore, it is important to find ways to foster collaboration between partners, such as supporting mutual respect and equality and cultivating shared goals and clearly defined roles and responsibilities for the various partners. It is also important to ensure that the research site reaps worthwhile benefits in return for participating in the research.

Ensuring that clinical research provides social value is an important precondition for ethical clinical research involving human subjects and, in order to achieve this, the research partners need to collaborate and share responsibility throughout the research process. Accordingly, more research is needed to clarify how social value is best defined, evaluated, measured and brought about in clinical research. There is also a need for greater transparency of clinical research activity and this means that the impact and benefits of clinical research must be demonstrated more efficiently, with respect to the population’s health, healthcare and society. The medical research community, researchers and senior hospital managers all need to play a key role in this.
